# Evaluation of large language model performance in translating dairy-related content

**DOI:** 10.1093/tas/txag130

**Published:** 2026-08-27

**Authors:** Anay D Ravelo, Daniela G Carranza, Kaitlyn A Lutz, Wolfgang Heuwieser

**Affiliations:** Department of Population Medicine and Diagnostic Sciences, Cornell University, Ithaca, NY 14850, United States; Cornell Cooperative Extension, Cornell University, Ithaca, NY 14850, United States; Cornell Cooperative Extension, Cornell University, Ithaca, NY 14850, United States; Department of Population Medicine and Diagnostic Sciences, Cornell University, Ithaca, NY 14850, United States

**Keywords:** artificial intelligence, English, Spanish, stockmanship, udder health

## Abstract

Artificial intelligence’s ability to translate dairy-related texts from English to Spanish has not been well described in agriculture. This study aimed to determine the accuracy and comprehensibility of dairy-related translated text generated with ChatGPT (GPT-4o) and to determine the text’s appropriateness for a dairy employee. Eight dairy udder health and stockmanship-related English texts were gathered from extension and university websites for translation. Four texts were summaries of 260 words or less and the other four texts were procedure lists with 8 steps. The texts were translated with a large language model (ChatGPT-4o) and displayed in a side-by-side presentation. The presentations were sent to 23 reviewers with a rubric for evaluation of accuracy, comprehensibility, and perception of the translation. Additionally, the reviewers were asked to evaluate if the translations were suitable to be shared with dairy employees. Overall, reviewers “Strongly agreed” or “Agreed” that the texts were accurately translated, including technical terms, and that the main idea of the text was correctly reproduced. They also “Strongly agreed” or “Agreed” that the translation was easy to understand, flowed naturally, was clear and coherent, and there was consistent translation of phrases that were repeated. Additionally, reviewers believed that there were “None” or “Few” instances where the meaning of the text is unclear due to the translation. Finally, the reviewers mostly agreed that they would present the translations to dairy employees. Thus, ChatGPT (GPT-4o) can accurately and comprehensibly translate dairy-related text. However, it is important to still have oversight and review the output.

## Teaser

After bilingual reviewers evaluated dairy related texts that were translated from English to Spanish using ChatGPT, it was considered that the texts were accurate, comprehensible, and precise. Thus, AI can be used to translate English content to Spanish for Spanish-speaking employees.

## Introduction

As herd sizes grow and demand for dairy products increases, U.S. dairy farms rely more heavily on non-family labor (Adcock et al. 2015). A large proportion of these employees are native Spanish speakers, whereas management is typically English-speaking. This language barrier can hinder effective communication and limit training success. Although tasks may be demonstrated, employees often lack the context to understand the rationale behind procedures. Without this understanding, it is difficult to build knowledge, improve skill retention, or foster a sense of professional growth. Consequently, annual employee turnover remains high, ranging from 39% to 144% (Durst et al. 2018), with two-thirds of workers citing a lack of development opportunities as their reason for leaving (Moore et al. 2020).

Historically, dairy farm owners and managers have relied on Spanish-speaking intermediaries to translate instructions and communicate with employees. The emergence of large language models (LLM), such as ChatGPT, now offers a potential alternative. These tools can translate text rapidly and may facilitate communication and protocol translation without external support. However, the accuracy and clarity of AI-generated translations in the context of dairy technical language have not yet been systematically evaluated.

Language barriers are also recognized in human medicine, where several studies have evaluated the translation performance of AI tools. With oversight from certified translators, AI-generated translations were found to be efficient and showed potential to improve healthcare access and bridge the literacy gap (Grimm et al. 2024; Brandenberger et al. 2025; Scheinkman et al. 2025). In studies focused on kidney donor education, AI was able to provide accurate and culturally appropriate information (Garcia Valencia et al. 2024, 2025). Similarly, in a study assessing Spanish AI-generated translations of dermatology materials, reviewers approved their use in four out of five topics (Dzuali et al. 2024).

The aim of this study is to gather data to help explore if an LLM can be used as a viable tool to translate dairy related text and technical language. This study did not conduct a head-to-head comparisons among ChatGPT variants. Thus, the objective of this study was to evaluate the quality of AI-generated translations from English to Spanish conducted with a large language model (LLM, GPT-4o, OpenAI, San Francisco, CA) of two types of dairy-related content (ie summaries of relevant topics and step-by-step task descriptions). Specifically, we set out (1) to evaluate the accuracy and comprehensibility of the translations, (2) to assess whether evaluators believed that the translations were appropriate for dairy employees.

## Materials and methods

A description of this research was submitted to the Cornell University Institutional Review Board and determined “to not be human participant research as the observational units for this study were the texts that were being translated.” Four summaries and four step-by-step task descriptions were adapted from milk quality extension materials available online through university websites. The summaries provided foundational knowledge and emphasized the importance of best practices related to milking routines, hygiene, milk sampling, and stockmanship. Topics addressed included the rationale for collecting clean milk samples, key principles of milking hygiene, an overview of proper milking procedures, and recommendations for moving cows efficiently into the parlor. The procedure lists offered step-by-step instructions for collecting a clean milk sample, administering intramammary antibiotics, conducting the milking routine, and guiding cow movement to the parlor. The English summaries had a similar text length of 252, 245, 241, and 260 words and each procedure list was limited to 8 steps (Supplementary file 1, available as supplementary data  *Translational Animal Science* online). The English texts were translated to Spanish using ChatGPT 4o on 13 May 2025 with a prompt that specified the purpose of the translation for educational use and the target audience of Hispanic dairy employees (Supplementary file 2, available as supplementary data  *Translational Animal Science* online). ChatGPT 4o was used since it was readily available to the research team at the time the study was conducted. However, the authors acknowledge that the use of other versions of ChatGPT may yield different results.

The structured prompt was the following: “Role: You are a bilingual dairy educator specializing in English to Spanish translations. Your task is to translate the following dairy related English text into Spanish. Ensure that the translation maintains the original meaning and is appropriate for Hispanic dairy employees. Task: Produce a high-quality Spanish translation that preserves the original nature of the English text. (1) Purpose of the Translation: The translation is for educational use. It should maintain the tone of the English text. (2) Target Audience: Hispanic dairy employees will read the translated text. They are native Spanish speakers and their highest education level is middle or high school.” Each English text that needed to be translated was pasted below this structured prompt in the command line.

For the distribution of English texts and their Spanish translations, a cloud-based presentation software (https://pitch.com/) was used to create four presentations. Each presentation consisted of one introduction page, one summary and its translation, and one step-by-step task description and its translation. Each presentation was accessible through a unique link. The use of the pitch decks enabled the Spanish AI-translated text and procedure lists to be presented alongside the original English texts and procedures. A 12-item rubric was developed to assess the translation, ensuring consistency, transparency, and a streamlined review process (Supplementary file 3, available as supplementary data  *Translational Animal Science* online). The rubric included the topics of accuracy, comprehensibility, and perception with 3, 4, and 1 statements/question to be answered on 4-point Likert scales. After completing the statements, reviewers were asked four questions: “What is your Spanish proficiency?”, “Would you provide this translation to a dairy employee?”, “Do you have any comments about the translation, such as any words or phrases that you find unusual?”, and “How much time did it take you to complete the rubric?”. The rubric was created by adapting the questions from medical studies that have considered the accuracy, comprehension, and precision of a translation (Dzuali et al. 2024; Scheinkman et al. 2025) into the context of dairy. A preliminary survey was conducted where two English dairy-related texts were translated into German, and three German speakers used the rubric to evaluate the texts. They deemed the rubric appropriate to assess the acceptability of the text.

The investigators identified potential reviewers through the Cornell University Cooperative Extension and Quality Milk Production Services. Eligibility criteria included fluency in both English and Spanish (at least at an intermediate level) and some prior experience working with dairy farms. A total of 38 individuals were identified and contacted via email, which included an introduction to the study and an invitation to participate (Supplementary file 4, available as supplementary data  *Translational Animal Science* online). Of those invited, 23 (60.5%) agreed to review the translated materials. Five distribution groups were created. Reviewers were assigned sequentially to the groups according to the order in which they confirmed participation, with every sixth reviewer restarting the assignment cycle in group 1. Within each group, a random number generator determined the order in which reviewers received the presentation links.

Within each distribution group, the order in which the four translation pairs with their unique links were presented to reviewers was determined using the random number generator function in Microsoft Excel. This approach was used to balance the number of responses across the four summaries and step-by-step task instructions.

Each reviewer then received an email containing the four translation links in their assigned sequence, along with the evaluation rubric. Reviewers were instructed to complete and return eight rubrics—one for each translation—within one month of receiving the initial email. To improve response rates, reminder emails were sent at two, three, and four weeks later if the rubrics had not been returned. Two weeks after receiving all eight completed rubrics, reviewers were contacted again and invited to re-evaluate one of the translations to assess intra-reviewer agreement. This follow-up email included the same link that had been presented first in their original set of four and specifically requested that reviewers not refer back to their initial evaluations.

All statistical analysis were performed using R studio (https://www.r-project.org/). Prior to contacting reviewers, a power analysis was conducted using the kappa variance formula. For a kappa of 0.7 with a variance of 0.15, an alpha of 0.05, and 8 texts used for evaluation we needed 14 observers to evaluate the texts. For comparison of the likert scale answers for each question an ordinal model was considered which had the fixed effects of text ID and the random effect of reviewer. To consider if reviewers would recommend the translations to a dairy employee a mixed linear model with the fixed effects of text ID and the random effect of reviewer was run using a generalized linear model considering the binomial. If the fixed effect of text ID was observed to be significant (*P* ≤ 0.05) then the model was stratified by text type to separate the models into summary and procedure lists. The kappa statistic for intra-reviewer agreement was calculated using the kappa2 function for the weighted kappa.

To identify and organize common themes in the feedback from reviewers ChatGPT -4o was used on 26 June 2025. The same prompt was used to organize the comments from the summaries and the procedures (Supplementary file 2, available as supplementary data  *Translational Animal Science* online) and after all comments were organized, a summary was requested with the following prompt: “Summarize all the key take aways from all of the 8 sets of comments.”

## Results

Out of the 23 reviewers who agreed to complete the translation reviews, 15 (65.2%) completed all of them, and 1 completed only 4 reviews; the others did not finish the review. Thus, summaries 1, 3, and 7, and procedure list 2 had 16 reviews, while summary 5 and procedure lists 4, 6, and 8 had 15 reviews (Table 1). Overall, for the statements considering accuracy and comprehensibility most reviewers answered “Strongly agree” and “Agree” for most of the texts. The two texts that considered stockmanship in the parlor (ie summary 7 and procedure list 8) received the most “Disagree” ratings. Summary 7 was the only text to receive a “Strongly disagree” observation for one of the accuracy questions. For the perception question, most reviewers selected “None” or “Few” instances where the meaning of the text was unclear due to the translation. Again summary 7 and procedure list 8 were the only ones to receive a review of “Several” and “Many” instances where the meaning of the text was unclear due to the translation.

**Table 1:** Reviewer responses by text type to rubric questions assessing the appropriateness of ChatGPT-4o Spanish translations of English dairy-related texts.

	Text ID							
Item	Summary 1	Summary 3	Summary 5	Summary 7	Procedure 2	Procedure 4	Procedure 6	Procedure 8
**Questions evaluated**	16	16	15	16	16	15	15	15
**Accuracy**								
** The text is correctly translated**								
** Strongly agree**	9 (56.2)	10 (62.5)	5 (33.3)	9 (56.2)	9 (56.2)	10 (66.7)	9 (60.0)	6 (40.0)
** Agree**	7 (43.8)	6 (37.5)	9 (60.0)	4 (25.0)	7 (43.8)	5 (33.3)	6 (40.0)	7 (46.7)
** Disagree**	0 (0.0)	0 (0.0)	1 (6.7)	3 (18.8)	0 (0.0)	0 (0.0)	0 (0.0)	2 (13.3)
** The technical terms related to dairy domain are correctly translated**								
** Strongly agree**	7 (43.8)	8 (50.0)	4 (26.7)	6 (37.5)	7 (43.8)	7 (46.7)	7 (46.7)	5 (33.3)
** Agree**	9 (56.2)	7 (43.8)	9 (60.0)	8 (50.0)	8 (50.0)	8 (53.3)	7 (46.7)	8 (53.3)
** Disagree**	0 (0.0)	1 (6.2)	2 (13.3)	1 (6.2)	1 (6.2)	0 (0.0)	1 (6.7)	2 (13.3)
** Strongly disagree**	0 (0.0)	0 (0.0)	0 (0.0)	1 (6.2)	0 (0.0)	0 (0.0)	0 (0.0)	0 (0.0)
** The main idea of the English text is correctly reproduced**								
** Strongly agree**	12 (75.0)	14 (87.5)	8 (53.3)	10 (62.5)	12 (75.0)	12 (80.0)	9 (60.0)	9 (60.0)
** Agree**	4 (25.0)	2 (12.5)	7 (46.7)	3(18.8)	4 (25.0)	3 (20.0)	6 (40.0)	5 (33.3)
** Disagree**	0 (0.0)	0 (0.0)	0 (0.0)	3(18.8)	0 (0.0)	0 (0.0)	0 (0.0)	1 (6.7)
**Comprehensibility**								
** The translation is easy to understand**								
** Strongly agree**	12 (75.0)	12 (75.0)	8 (53.3)	9 (56.2)	12 (75.0)	11 (73.3)	8 (53.3)	8 (53.3)
** Agree**	4 (25.0)	4 (25.0)	6 (40.0)	4 (25.0)	4 (25.0)	3 (20.0)	7 (46.7)	4 (26.7)
** Disagree**	0 (0.0)	0 (0.0)	1 (6.7)	3(18.8)	0 (0.0)	1 (6.7)	0 (0.0)	3 (20.0)
** The language flows naturally**								
** Strongly agree**	9 (56.2)	12 (75.0)	8 (53.3)	9 (56.2)	10 (62.5)	9 (60.0)	8 (53.3)	7 (46.7)
** Agree**	6 (37.5)	4 (25.0)	7 (46.7)	3(18.8)	5 (31.2)	6 (40.0)	7 (46.7)	5 (33.3)
** Disagree**	1 (6.2)	0 (0.0)	0 (0.0)	4 (25.0)	1 (6.2)	0 (0.0)	0 (0.0)	3 (20.0)
** The text is clear and coherent**								
** Strongly agree**	10 (62.5)	11 (68.8)	8 (53.3)	8 (50.0)	8 (50.0)	7 (46.7)	9 (60.0)	8 (53.3)
** Agree**	6 (37.5)	5 (31.2)	7 (46.7)	4 (25.0)	8 (50.0)	8 (53.3)	5 (33.3)	3 (20.0)
** Disagree**	0 (0.0)	0 (0.0)	0 (0.0)	4 (25.0)	0 (0.0)	0 (0.0)	1 (6.7)	4 (26.7)
** There is consistent translation of phrases that come up repeatably**								
** Strongly agree**	9 (56.2)	10 (62.5)	5 (33.3)	6 (37.5)	10 (62.5)	8 (53.3)	8 (53.3)	7 (46.7)
** Agree**	7 (43.8)	6 (37.5)	9 (60.0)	7 (43.8)	6 (37.5)	7 (46.7)	7 (46.7)	7 (46.7)
** Disagree**	0 (0.0)	0 (0.0)	1 (6.7)	3 (18.8)	0 (0.0)	0 (0.0)	0 (0.0)	1 (6.7)
**Perception**								
** Are there instances where the meaning of the text is unclear due to the translation?**								
** None**	11 (68.8)	11 (68.8)	7 (46.7)	9 (56.2)	10 (62.5)	7 (46.7)	8 (53.3)	8 (53.3)
** Few**	5 (31.2)	5 (31.2)	8 (53.3)	4 (25.0)	6 (37.5)	8 (53.3)	7 (46.7)	5 (33.3)
** Several**	0 (0.0)	0 (0.0)	0 (0.0)	2 (12.5)	0 (0.0)	0 (0.0)	0 (0.0)	1 (6.7)
** Many**	0 (0.0)	0 (0.0)	0 (0.0)	1 (6.2)	0 (0.0)	0 (0.0)	0 (0.0)	1 (6.7)
**Time, average (*SD*)**								
** How much time did it take you to complete the rubric?**	9.9 (6.7)	9.6 (5.4)	8.9 (4.7)	12.4 (8.6)	9.3 (7.3)	7.6 (3.8)	7.3 (4.2)	8.5 (4.0)

For the accuracy statement of “The text is correctly translated” there were no difference based on the text types (*P* = 0.19; Table 2). Similarly, for the statement of “The technical terms related to the dairy domain are correctly translated” no differences were detected based on text (*P* = 0.52; Table 2). For the statement of “The main idea of the English text is correctly reproduced,” no differences were detected for text ID (*P* = 0.08; Table 2).

**Table 2:** The LS means and SEM of reviewer responses analyzed using a generalized linear mixed model considering the logit link function to assess the appropriateness of ChatGPT-4o Spanish translations of English dairy-related texts.

	Text ID									
	Summary 1	Summary 3	Summary 5	Summary 7	Procedure 2	Procedure 4	Procedure 6	Procedure 8	SEM	*P*-value
**Questions evaluated**	16	16	15	16	16	15	15	15		
**Accuracy**										
** The text is correctly translated**	−2.56	−2.92	−1.20	−1.83	−2.57	−3.11	−2.71	−1.14	0.79	0.19
** The technical terms related to dairy domain are correctly translated**	−3.48	−3.58	−2.10	−2.66	−3.23	−3.63	−3.35	−2.42	0.86	0.52
** The main idea of the English text is correctly reproduced**	−5.15	−6.56	−3.09	−3.02	−5.15	−5.39	−3.55	−3.20	1.40	0.08
**Comprehensibility**										
** The translation is easy to understand**	−5.36	−5.34	−3.23	−2.18	−5.36	−4.45	−3.41	−2.13	1.47	0.02
** The language flows naturally**	−3.16	−5.24	−2.78	−1.97	−3.61	−3.80	−3.27	−1.51	1.21	0.08
** The text is clear and coherent**	−3.55	−3.99	−2.45	−1.49	−2.75	−2.41	−2.98	−1.39	1.03	0.17
** There is consistent translation of phrases that come up repeatably**	−3.82	−4.31	−1.43	−1.21	−4.32	−3.36	−3.36	−2.42	1.14	0.08
**Preception**										
** Are there instances where the meaning of the text is unclear due to the translation?**	−4.89	−4.87	−3.64	−3.11	−4.46	−3.64	−4.02	−3.25	1.01	0.41

For the comprehensibility statement of “The translation is easy to understand” the text was significant (*P* = 0.02; Table 2) and the model was stratified by text type ie whether summary or procedure. When considering the summary texts there was no difference among them (*P* = 0.26). Likewise for the procedure texts there was no difference detected among texts (*P* = 0.20). For “The language flows naturally” statement there were no differences detected by text (*P* = 0.08; Table 2). Similarly, for “The text is clear and coherent” statement there were no differences detected by text (*P* = 0.17; Table 2). For the final comprehensibility statement “There is consistent translation of phrases that come up repeatably” there were no difference detected by text (*P* = 0.08; Table 2).

When considering the perception question of “Are there instances where the meaning of the text is unclear due to the translation?” there were no differences detected by text (*P* = 0.41; Table 2). When evaluating whether the reviewer would give this translation to a dairy employee, no differences were detected by the text (*P* = 0.86). For summary 1 and 3, 16 reviewers (100%) recommended the translation for dairy employees. For summary 5 and 7, 12 reviewers recommended the translation to dairy employees, which would be 80% and 75% of the reviewers, respectively. Procedure list 2 and 6 were recommended by 14 reviewers, which would be 87.5% and 93.3% of the reviewers, respectively. Procedure list 4 was recommended by 13 reviewers (86.7%) and procedure list 8 was recommended by 12 reviewers (80.0%).

For the intra-reviewer analysis of the 16 individuals who submitted rubrics, 8 individuals completed the follow-up evaluation and submitted rubrics for two evaluations they had previously completed. The average intra-reviewer agreement or weighted kappa was 0.64 with a range of 0.29 to 1. Overall, when feedback comments were considered, reviewers believed that the core message of the translations was understandable; however, there were several instances where the translation was identified as being literal or to have technical inconsistencies. At least three reviewers perceived and commented that the literal translation disrupted the flow and readability of the text in Spanish. Reviewers also believed that sometimes the translation would be too specific and complex, which would not be ideal for employees with lower literacy levels. Other concerns with the translation was that dairy specific terms such as “forestrip” and “crowd gate” were not translated to the commonly used terms (“despuntar” and “puerta de acoplamiento, porton, and/or puerta de empuje”) in Spanish. There was also some concern that different geographical regions would use different terminology than the ones used by ChatGPT. Additionally, there was at least one instance observed where the translation used the wrong word, for example it translated “milk them” to “organize them” in Spanish. This may have been due to a similar spelling in Spanish, but the error would change the meaning of the text.

## Discussion

Although AI has the ability to translate text from English to a desired language, its ability to translate dairy-related text from English to Spanish accurately has not been well described. The current study demonstrated that bilingual reviewers were impressed with the translations generated by ChatGPT-4o when asked to evaluate AI-translated text. When considering accuracy and comprehensibility of the translated text many replied on the positive end of the Likert scale with selections such as “Strongly agree” and “Agree.” When asked about text clarity, most respondents selected “None” or “Few” unclear parts. The majority also indicated they would share the translation with a dairy employee. Thus, ChatGPT 4o can be used to accurately translate dairy-related text from English to Spanish.

This study aimed at maintaining consistency by using a single pre-defined prompt for all translations. When Andalib (2025) compared different prompts and how they influence AI’s ability to translate orthopedic medical text to Spanish, their findings suggested that prompt optimization may improve translation outcomes. The authors speculate that if more specific information had been included in the prompt, the translations could have potentially been improved. Future work could benefit from refining prompt design to better match the linguistic and contextual needs of veterinary and medical domains. For example, prompts could be enhanced by specifying preferred technical terminology, indicating whether regional variants of Spanish should be used, and providing contextual cues related to the target audience or use case. Such refinements may increase both the accuracy and practical relevance of translations, particularly when conveying specialized concepts that require precision. Further development of large language models themselves may also improve the quality of AI-generated translations, especially in specialized technical fields.

In two previous studies conducted in human medicine AI-generated translations were reviewed by only two native Spanish-speakers that were experts in the fields of nephrology and oncology (Garcia Valencia et al. 2024; Rodler et al. 2025). The current study included 14 reviewers who had more diverse experience with Spanish proficiency (Intermediate, Fluent, and Native). It is possible that Spanish proficiency and difference in experience may influence how reviewers perceive text; however, our study was not designed to test this.

Scheinkman et al. (2025) evaluated AI’s ability to translate surgical instructions. They observed that DeepL had an overall sentence accuracy value of 96.6% and an overall comprehensibility value of 95.9%. Thus, they concluded that with appropriate oversight by medical translators AI could be able to help translate surgical-related text from English to Spanish. This study analyzed individual words and sentences to measure accuracy and medical terms to consider comprehensibility, while the current study used statement agreement for measurements of accuracy and comprehensibility. Overall, 81.2% of the reviewers agreed with the accuracy statements and 73.3% reviewers agreed with the comprehensibility statements. These findings align with Scheinkman et al. (2025), suggesting that AI can effectively translate dairy-related texts with the validation of a Spanish-speaker who is familiar with the dairy industry.

When reviewers were asked to evaluate misinformation and errors in dermatology-related texts that had been translated from English to Spanish using Chat GPT it was observed that most texts had less than 3 instances of misinformation and between 3–10 instances of grammatical and spelling errors (Dzuali et al. 2024). In the current study, reviewers were asked only whether “there are instances where the meaning of the text is unclear due to the translation,” with the response options “None,” “Few,” “Several,” or “Many.” Most reviewers selected “None” or “Few.” Overall, the perception of the texts was positive and thus AI can be used to translate dairy-related text from English to Spanish. Such translations can be further improved if they are validated and proofread by a Spanish speaking expert in the field. Similarly, Dzuali et al. (2024) reported that most reviewers would provide the Spanish translation to a patient aligning to the current study where most texts were considered suitable for a dairy employee.

When reviewers re-evaluated the translations they had previously assessed, the intra-reviewer agreement was 0.64 suggesting that their perceptions and evaluations remained relatively consistent over time. When considering if they would provide the translation to a dairy employee, out of 16 responses 13 agreed with the original selection. Garcia Valencia et al. (2024) observed that the two reviewers had an inter-reviewer agreement of 0.85. The inter-reviewer agreement was not calculated in the current study due to the greater number of reviewers in the study. It is likely, however, that it would not have been as high since the reviewers that were evaluated in Garcia Valencia et al. (2024) had more similar education and experiences as they were both native Spanish-speaking nephrologists of Mexican heritage.

LLMs can translate dairy-related materials into Spanish. This capability enables English-speaking managers, extension personnel, and veterinarians to translate SOPs, protocols, and peer-reviewed educational resources. Translation of proprietary documents should occur only with the owner’s permission. Although LLMs can generate training content de novo, translating established materials from reputable sources may be preferable because these documents have typically undergone expert peer review and are regularly updated.

Limitations of this study include that we had a limited number of texts, we focused only on the topic of udder health and stockmanship, and it was a convenience sample of reviewers that were identified through connections with the research team. To not overwhelm reviewers, we tried to limit the number of texts, words, and procedures in the translations which limited the amount of dairy-related terminology. We also had a limited number of reviewers and lost half of them during the follow-up period which could introduce bias into the results, thus future studies should consider more reviewers. Furthermore, most texts focused on udder health, limiting the generalizability of these findings to other dairy-related topics. Additionally, all texts were translated using a single structured prompt, which prevented assessment of if a standard or optimized prompt might affect AI translation accuracy. Future studies should consider the impact of improving prompt structure by specifying terminology, regional variants, and contextual details since this was outside of the scope of this study. Finally, this study was not designed to test the performance of specialized translation tools (eg Google Translate, DeepL), thus the results of this study can only support the feasibility of using LLM for translations and not its performance compared to specialized machine translation systems. Moreover, the current study did not evaluate model families of ChatGPT and the results cannot be interpreted as evidence that GPT-4o out performs other model versions. Additionally, the results are model and version specific and likely cannot be generalized to other variants without direct testing.

## Conclusion

ChatGPT (GPT-4o) can produce accurate and comprehensible translations of dairy-related texts. These translations are appropriate for Spanish-speaking employees. However, there were instances where the meaning of the text was unclear due to the translation. Therefore, we encourage having the output evaluated by Spanish-speaking individuals.

## Supplementary Material

txag130_Supplementary_Data

## List of abbreviations

AI: Artificial Intelligence
LLM: Large Language Models
