# Supplementary material for: Evaluation of large language model performance in translating dairy-related content
Source: Transl Anim Sci. 2026 Aug 27;10:txag130. doi: 10.1093/tas/txag130 (PMC13553073; doi:10.1093/tas/txag130)
Supplement: Supplementary file 1 [file txag130_supplementary_data.zip › Supplementary file 3.docx]

**How well did AI translate the text?**

Text#:_____________________

| **Accuracy** | Strongly agree | Agree | Disagree | Strongly disagree |
| --- | --- | --- | --- | --- |
| The text is correctly translated | ☐ | ☐ | ☐ | ☐ |
| The technical terms related to dairy domain are correctly translated | ☐ | ☐ | ☐ | ☐ |
| The main idea of the English text is correctly reproduced | ☐ | ☐ | ☐ | ☐ |
| **Comprehensibility** | Strongly agree | Agree | Disagree | Strongly disagree |
| The translation is easy to understand | ☐ | ☐ | ☐ | ☐ |
| The language flows naturally | ☐ | ☐ | ☐ | ☐ |
| The text is clear and coherent | ☐ | ☐ | ☐ | ☐ |
| There is consistent translation of phrases that come up repeatably | ☐ | ☐ | ☐ | ☐ |
| **Perception** | None | Few | Several | Many |
| Are there instances where the meaning of the text is unclear due to the translation? | ☐ | ☐ | ☐ | ☐ |

What is your Spanish proficiency?

☐ Native speaker

☐ Fluent

☐ Intermediate

☐ Beginner

Would you provide this translation to a dairy employee?

☐ Yes

☐ No

Do you have any comments about the translation, such as any words or phrases that you find unusual?

How much time did it take you to complete the rubric?
