# Supplementary material for: Evaluation of large language model performance in translating dairy-related content
Source: Transl Anim Sci. 2026 Aug 27;10:txag130. doi: 10.1093/tas/txag130 (PMC13553073; doi:10.1093/tas/txag130)
Supplement: Supplementary file 1 [file txag130_supplementary_data.zip › Supplementary file 4.docx]

Hello Potential Reviewer,

My name is Anay Ravelo. I am a postdoc with Quality Milk Production Services. XXX identified you as a potential reviewer in the following study aimed to evaluate the ability of AI to translate texts from English to Spanish.

**Question:** Is AI able to translate dairy related texts from English to Spanish correctly?

**Design:** You will be sent a rubric and links. The links will lead to a pitch site which will have three pages:

1. Introduction page with a description of the study and task.
2. One English dairy related summary and its AI generated Spanish translation.
3. One English dairy related procedure list and its AI generated Spanish translation.

**Your task:** We kindly ask that you evaluate each translation using the provided rubric. In total it will probably take about 10-15 minutes to complete the rubric for each link. We acknowledge that the summaries and procedure lists may not be based on the most current evidence. Therefore, we ask that you focus your attention on the quality and appropriateness of the translation to be presented to a dairy employee and not on if content is current or outdated.

Ideally, you would agree to review 4-5 links. The total time investment is expected to be around 60-75 minutes for the whole study. Participants who are willing to review links will receive a $20 dollar gift card after the review process is complete.

Once the links are shared with you, you will have one month to send back the completed reviews. This is for a study that we plan to publish if the results are adequate. If you are interested, we can share a summary of the results with you.

If you would like to participate, please click the link below.

“Link”

Once you agree to participate you will receive the links within 3 days. We look forward to hearing from you! Thank you for considering.

If you have any further questions, feel free to reach out to me.

Best,

Anay
