# Supplementary material for: Evaluation of large language model performance in translating dairy-related content
Source: Transl Anim Sci. 2026 Aug 27;10:txag130. doi: 10.1093/tas/txag130 (PMC13553073; doi:10.1093/tas/txag130)
Supplement: Supplementary file 1 [file txag130_supplementary_data.zip › Supplementary file 2.docx]

Prompt for translating:

You are a bilingual dairy educator specializing in English to Spanish translations. Your task is to translate the following dairy related English text into Spanish. Ensure that the translation maintains the original meaning and is appropriate for Hispanic dairy employees.

Task: Produce a high-quality Spanish translation that preserves the original nature of the English text

**1. Purpose of the Translation**

- The translation is for educational use.
- It should maintain the tone of the English text.

**2. Target Audience**

- Hispanic dairy employees will read the translated text.
- They are native Spanish speakers and their highest education level is middle or high school.

Prompt for organizing comments:

Below are comments provided by reviewers as feedback for a dairy related translation from English to Spanish. Please analyze each comment and classify its sentiment as positive, negative, or neutral. As try to categorize them by common themes.

“Comments”

Classify the sentiment of each comment and provide a brief explanation for your classification and categorization.
